# Supplementary material for: Knockdown resistance (kdr) associated organochlorine resistance in mosquito-borne diseases (Culex quinquefasciatus): Systematic study of reviews and meta-analysis
Source: PLoS Negl Trop Dis. 2024 Aug 19;18(8):e0011991. doi: 10.1371/journal.pntd.0011991 (PMC11361747; doi:10.1371/journal.pntd.0011991)
Supplement: Attachment — Submitted filename: Replay to eviewer .docx [file pntd.0011991.s002.docx]

**Dear Editor-in-Chief of PLOS Neglected Tropical Diseases**

**Greetings and Regards**

**Thank you for taking the time to review my manuscript.**

**The answers of the Reviewers have been sent to your service attachment. I hope that is convincing.**

| Reviewer | Comment | Response |
| --- | --- | --- |
| Reviewer 1 | 1. kdr should be italicized | Done |
| Reviewer 1 | 2. It is well known that pyrethroids are currently the most commonly used insecticides. Why are you researching organochlorine insecticides? You need to elaborate on this. | Organochlorine insecticides have been used since ancient times to combat Anopheles. Recently, some studies have shown that due to the lack of long-term use of organochlorines, the sensitivity of some Anopheles species to them has increased and it may be possible to use them again. Although the environmental hazards of these insecticides still persist.  However, one of the main goals of this study was to investigate the prevalence of KDR in relation to organochlorine toxins and compare it with other insecticides to determine the resistance ratio in them and to pay attention to this issue during the implementation of vector control programs. |
| Reviewer 1 | 3. Organochlorine resistance is often associated with metabolic resistance, while pyrethroids are often linked to kdr. Why have you chosen to study Knockdown resistance (kdr) Associated | Why knockdown resistance is checked instead of metabolic resistance in Organochlorine toxins  In the context of studying resistance mechanisms to organochlorine pesticides, knockdown resistance (kdr) is often prioritized over metabolic resistance due to several reasons rooted in the mechanisms of action, ease of detection, and historical context.  1. Mechanism of Action:  o Knockdown Resistance: Organochlorine pesticides, such as DDT, primarily target the sodium channels in the nervous systems of insects. Knockdown resistance occurs due to mutations in these sodium channels, rendering the insect less sensitive to the pesticide. This form of resistance is directly linked to the primary mode of action of the pesticide, making it a crucial point of investigation.  o Metabolic Resistance: Metabolic resistance involves the enhancement of detoxification pathways, such as increased activity of enzymes like cytochrome P450 monooxygenases, glutathione S-transferases, or esterases, which metabolize the pesticide into less toxic forms. Although important, metabolic resistance represents a more generalized and less specific mechanism compared to kdr.  2. Ease of Detection:  o Knockdown Resistance: Detecting kdr is often more straightforward because it involves identifying specific genetic mutations in the sodium channel gene. Molecular techniques such as PCR (polymerase chain reaction) and sequencing can efficiently detect these mutations, providing a clear and direct measure of resistance.  o Metabolic Resistance: Assessing metabolic resistance is more complex, as it requires measuring the activity levels of various detoxifying enzymes, which can be influenced by multiple factors. This often involves bioassays, biochemical assays, and sometimes advanced analytical techniques like mass spectrometry, which are more resource-intensive and time-consuming.  3. Historical Context:  o The study of knockdown resistance has a historical precedent because the phenomenon was first observed and characterized in response to DDT and other organochlorine insecticides. The term "kdr" was coined to describe this specific form of resistance, which led to a focused research effort on understanding and detecting these mutations.  4. Direct Impact on Efficacy:  o Knockdown Resistance: Mutations causing kdr directly impact the efficacy of the pesticide, leading to an immediate reduction in the knockdown effect and overall mortality of the insect population. Monitoring kdr provides direct insights into the potential failure of control measures.  o Metabolic Resistance: While significant, metabolic resistance may not always lead to complete pesticide failure, as it can vary in efficiency and be partially overcome by increasing doses or using synergists.  Given these points, the emphasis on knockdown resistance in research and monitoring efforts allows for a more targeted, efficient, and historically grounded approach to understanding and managing resistance to organochlorine pesticides. |
| Reviewer 1 | 4. Line 140-148: The results simply list the mortality rates of Anopheles mosquitoes to various insecticides without indicating whether there is cross-resistance between different insecticides. | Explanations were added, although this study was conducted using a meta-analysis method and with quantitative data, and its purpose was to report prevalence. That prevalence data can be used for management planning, equipment supply and decision-making. |
| Reviewer 1 | 5. Line 150-154: Do Culex quinquefasciatus mosquitoes with different mortality rates to different insecticides have different kdr mutations? Do kdr mutations at different loci enhance or reduce insecticide resistance? The origin and evolutionary direction of kdr mutations are the main focus of analysis. | Explanations were added. |
| Reviewer 1 | 6. The discussion is merely a compilation of literature. For instance, the results do not indicate that kdr mutations are the most important resistance mechanism of insects against DDT, an o organochlorine There is a lack of in-depth analysis. | It was explained earlier in the discussion section. Some items were also added. |
